# Revolutionizing care: unleashing the power of comprehensive geriatric assessment in tailoring treatment for frail postintensive care patients

**DOI:** 10.62675/2965-2774.20240023-en

**Published:** 2024-05-07

**Authors:** João Gabriel Rosa Ramos, Michele Melo Bautista, Rafael Calazans, Luciulo Melo, Cassiano Teixeira

**Affiliations:** 1 Clínica Florence Salvador BA Brazil Clínica Florence - Salvador (BA), Brazil.; 2 Universidade Federal de Ciências da Saúde de Porto Alegre Internal Medicine Department and Rehabilitations Sciences Porto Alegre RS Brazil Internal Medicine Department and Rehabilitations Sciences, Universidade Federal de Ciências da Saúde de Porto Alegre - Porto Alegre (RS), Brazil.

Frailty represents a condition of vulnerability leading to inadequate recovery following a stressful event, such as an acute illness or injury. This inadequate recovery results from cumulative, multisystem physiological depletion over a lifetime.^(1)^ The frailty state implies that the available functional reserve is insufficient for complete recovery, often leading to a maladaptive response disproportionate to the degree of insult.^(2)^ Frailty syndrome comprises five core components: vulnerability to stressors, multifactorial etiology causing multisystem dysregulation, heterogeneous presentation, clinical measurability, and association with adverse outcomes.^(3)^ These components underscore frailty as a treatable clinical syndrome with a measurable biological basis.^(2)^

Importantly, frailty is separate from but related to older age, multimorbidity or disability. For example, up to 4% of adults less than 65 years of age are frail, and up to 38% are prefrail, with an increasing prevalence in multimorbid patients.^(4)^ Additionally, even though disability and comorbidities overlap with frailty, 8.6% of frail patients have no disabilities or comorbidities.^(5)^ Thus, while conventionally linked to older age and health issues, frailty is now recognized as a dynamic transitional state from robustness to functional decline, potentially preventable or reversible in some cases.^(2)^

The trajectory of critical illness closely aligns with the frailty process. Critical illness affects patients’ functional trajectory, with a substantial proportion of patients facing death or functional decline within a year after intensive care unit (ICU) admission. Worse outcomes are observed in patients with poorer premorbid functional status.^(6,7)^ Frailty may be present in up to 45% of patients 90 days after ICU discharge, with 46% transitioning to a worse frailty status.^(8)^ Additionally, 61% of patients who presented with frailty at follow-up were not frail at baseline, suggesting ICU-induced frailty.^(8)^ Furthermore, pre-ICU frailty correlates with adverse short-term and long-term outcomes in critically ill patients, irrespective of age.^(9-12)^ Hence, preexisting frailty affects the response to critical illness, but critical illness also influences the development and progression of frailty.

The bidirectional relationship between critical illness and frailty status can be explained by various factors. Critical illness may impair organ functions, exacerbate existing comorbidities or lead to new morbidities.^(13)^ Furthermore, critical illness may trigger biological aging processes, including cell senescence.^(14)^ Biological markers shared between critical illness and frailty may be involved in similar processes.^(2,13)^ Social and cognitive aspects of critical illness survivorship, such as cognitive decline, socioeconomic deprivation, social isolation, and lack of support, contribute to frailty development or progression.^(13,15)^

Intensive care unit survivors face the risk of developing postintensive care syndrome (PICS), characterized by the emergence or exacerbation of physical, psychological, cognitive, and mental health impairments, along with socioeconomic challenges.^(15)^ Post-ICU care models have emerged to address these issues, employing multidisciplinary recovery programs primarily grounded in outpatient clinic models. These models are based on a structured, multidisciplinary assessment of each impairment, with a focus on identifying and addressing disabilities; managing comorbidities, especially conditions that can lead to hospital readmissions, entailing a thorough review and adjustment of pharmacological therapies; and evaluating treatment burden, risks, social contexts, and health care contexts. This approach leads to a personalized strategy for therapies and discussions about the goals of care.^(13,15)^ Despite aligning with the recommendations for managing complex, multimorbid patients, there is a lack of robust data supporting the efficacy and cost-effectiveness of these models.^(13,15)^ The absence of standardized frailty assessments to better predict outcomes and guide treatment decisions could contribute to the dearth of positive results in studies.^(16)^ Indeed, there is evidence suggesting that, in using this conventional outpatient based model, more frail patients may face a heightened risk of unfavorable outcomes.^(17)^

In geriatrics, the Comprehensive Geriatric Assessment (CGA) is a multidisciplinary diagnostic process in which a coordinated plan for managing complex health care conditions and maximizing overall health is formulated.^(18)^ It has been shown to be effective for various conditions, such as emergency and orthopedic surgery; medical admissions; and health outcomes, such as falls, nursing home admission, pressure sores, delirium, and physical frailty.^(18)^ Utilizing structured tools,^(13, 15)^ a CGA requires a clinician with expertise and a multidisciplinary team, mirroring the current post-ICU care model.

**Figure 1:**
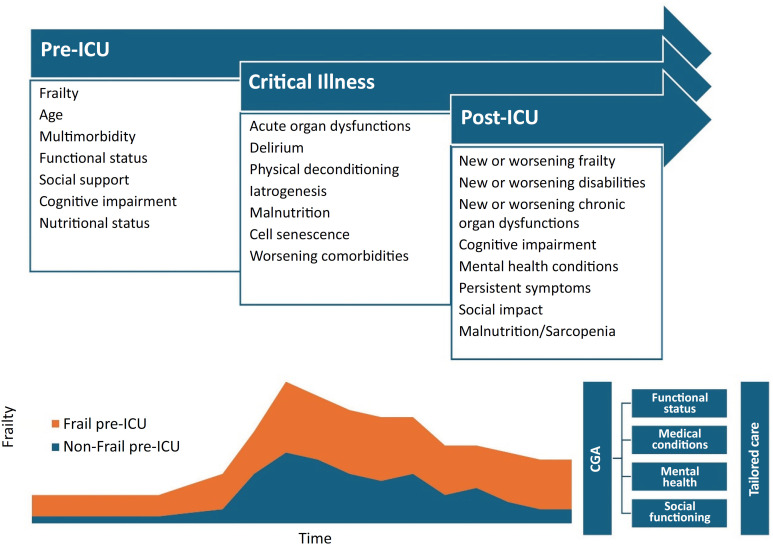
Clinical course of frailty in frail and nonfrail critically ill patients, with the proposed role of the Comprehensive Geriatric Assessment in the postintensive care unit model of care. ICU - intensive care unit; CGA - Comprehensive Geriatric Assessment.

Thus, the CGA process can be adapted to the post-ICU setting (Figure 1). The systematic use of the CGA in post-ICU care could benefit survivors through several mechanisms.^(13,15)^ First, by employing structured tools similar to those recommended for post-ICU care, CGA allows for a comprehensive and multidisciplinary evaluation of various domains. This structured approach facilitates the identification of frail patients who may require alternative models of care, such as admission to postacute care facilities or home-based health care, more intensive rehabilitation or increased follow-up frequency. This targeted approach, also called geriatric assessment-guided management (GAM), aims to minimize the risk of implementing inappropriate treatments and mitigates the potential for iatrogenic disease. Furthermore, the standardized, structured integration of information from multiple domains in the CGA process contributes to the development of an individualized management plan.

This personalized strategy enhances the overall quality of care by addressing the unique needs and complexities of each patient. Moreover, applying the CGA process to stratify post-ICU care based on frailty status enables the creation of tailored care models. This approach recognizes and addresses diverse health care conditions and needs, ultimately improving the effectiveness and responsiveness of post-ICU interventions. By adapting the CGA process to the post-ICU setting, health care providers can enhance the quality of care for ICU survivors, minimizing the potential negative outcomes associated with the PICS.

In summary, the systematic implementation of CGAs in post-ICU care has the potential to optimize patient outcomes by offering targeted interventions, personalized management plans, and tailored care models, thus contributing to the overall improvement of health care delivery for ICU survivors.
