# Supplementary material for: Revolutionizing care: unleashing the power of comprehensive geriatric assessment in tailoring treatment for frail postintensive care patients
Source: Crit Care Sci. 2024 May 7;36:e20240023en. doi: 10.62675/2965-2774.20240023-en (PMC11098071; doi:10.62675/2965-2774.20240023-en)
Supplement: Translation

# Translation: Revolucionando o cuidado: o poder da avaliação geriátrica ampla na personalização do tratamento de pacientes frágeis necessitando de assistência pós-unidade de terapia intensiva

**DOI:** 10.62675/2965-2774.20240023-pt

**Authors:** João Gabriel Rosa Ramos, Michele Melo Bautista, Rafael Calazans, Luciulo Melo, Cassiano Teixeira

**Affiliations:** 1 Salvador BA Brasil Clínica Florence - Salvador (BA), Brasil.; 2 Porto Alegre RS Brasil Departamento de Medicina Interna e Ciências da Reabilitação, Universidade Federal de Ciências da Saúde de Porto Alegre - Porto Alegre (RS), Brasil.

A fragilidade representa uma condição de vulnerabilidade que impede a recuperação adequada após um evento estressante, como uma doença ou lesão aguda. A recuperação inadequada resulta de uma depleção fisiológica cumulativa e multissistêmica ao longo da vida.^(1)^ O estado de fragilidade indica que a reserva funcional disponível é insuficiente para possibilitar uma recuperação completa, normalmente resultando em uma resposta inadequada e desproporcional ao grau de insulto.^(2)^ A síndrome da fragilidade compreende cinco componentes principais: vulnerabilidade a estressores, etiologia multifatorial causando desregulação multissistêmica, apresentação heterogênea, mensurabilidade clínica e associação a desfechos adversos.^(3)^ Esses componentes reforçam que a fragilidade é uma síndrome clínica tratável e que apresenta uma base biológica mensurável.^(2)^

É importante ressaltar que a fragilidade é uma característica independente, mas está relacionada com idade avançada, múltiplas morbidades e incapacidade. Por exemplo, até 4% dos adultos com menos de 65 anos são frágeis, e até 38% são pré-frágeis, com prevalência crescente em pacientes com múltiplas morbidades.^(4)^ Além disso, embora incapacidade e comorbidades possam ocorrer com a fragilidade, 8,6% dos pacientes frágeis não apresentam incapacidade nem comorbidades.^(5)^ Assim, embora convencionalmente ligada à idade avançada e a problemas de saúde, a fragilidade é atualmente reconhecida como um estado transitório dinâmico entre um estado saudável e um de declínio funcional, no qual a fragilidade é potencialmente prevenível ou reversível em alguns casos.^(2)^

A trajetória da doença crítica está intimamente alinhada com o processo de fragilidade. A doença crítica afeta a trajetória funcional dos pacientes, com proporção substancial de pacientes enfrentando morte ou declínio funcional dentro de 1 ano após internação na unidade de terapia intensiva (UTI). Desfechos piores são observados em pacientes com pior estado funcional pré-mórbido.^(6, 7)^ A fragilidade pode acometer até 45% dos pacientes 90 dias após a alta da UTI, com 46% progredindo para um estado de fragilidade pior.^(8)^ Brummel et al. apontam que 61% dos pacientes que apresentaram fragilidade no acompanhamento não eram frágeis no início do estudo, o que sugere que a fragilidade foi causada pela UTI.^(8)^ Além disso, a fragilidade pré-UTI está correlacionada com desfechos adversos a curto e longo prazo em pacientes críticos, independentemente da idade.^(9-12)^ Portanto, a fragilidade preexistente afeta a resposta à doença crítica, mas a doença crítica também influencia o desenvolvimento e a progressão da fragilidade.

A relação bidirecional entre doença crítica e estado de fragilidade pode ser explicada por vários fatores. A doença crítica pode prejudicar a função dos órgãos, exacerbar comorbidades existentes ou causar novas morbidades.^(13)^ Além disso, a doença crítica pode desencadear processos de envelhecimento biológico, incluindo senescência celular.^(14)^ Marcadores biológicos compartilhados entre doença crítica e fragilidade podem estar envolvidos em processos semelhantes.^(2, 13)^ Aspectos sociais e cognitivos da sobrevivência à doença crítica, como declínio cognitivo, privação socioeconômica, isolamento social e falta de apoio, contribuem para o desenvolvimento ou progressão da fragilidade.^(13, 15)^ Portanto, pacientes que sobrevivem à UTI têm risco maior de desenvolver fragilidade ou progressão da fragilidade e devem ser manejados adequadamente.

Os sobreviventes da UTI apresentam risco de desenvolver síndrome pós-cuidados intensivos (PICS - *postintensive care syndrome*), caracterizada pelo surgimento ou exacerbamento de comprometimento físico, psicológico, cognitivo e de saúde mental, com desafios socioeconômicos.^(15)^ Modelos assistenciais pós-UTI surgiram para abordar esses problemas, empregando programas de recuperação multidisciplinares baseados principalmente em modelos ambulatoriais. Esses modelos são baseados em uma avaliação estruturada e multidisciplinar de cada comprometimento, com foco na identificação e abordagem de incapacidades; no manejo de comorbidades, especialmente condições que possam levar a novas internações, envolvendo revisão e ajuste minuciosos das terapias farmacológicas; e avaliação da carga do tratamento, riscos, contextos sociais e contextos assistenciais. Essa abordagem promove uma estratégia personalizada para terapias e discussões sobre os objetivos do cuidado.^(13, 15)^ Apesar de estarem alinhados com as recomendações de manejo de pacientes complexos e com múltiplas morbidades, não há dados robustos que sustentem a eficácia e a relação de custo-efetividade desses modelos.^(13, 15)^ A ausência de avaliações padronizadas de fragilidade para melhor prever os desfechos e orientar as decisões de tratamento pode contribuir para a escassez de resultados positivos em estudos.^(16)^ Inclusive, há evidências que sugerem que, ao usar esse modelo baseado em modelos ambulatoriais, mais pacientes frágeis podem enfrentar um risco aumentado de desfechos desfavoráveis.^(17)^

Na geriatria, a avaliação geriátrica ampla (AGA) é um processo diagnóstico multidisciplinar, no qual é formulado um plano coordenado para o manejo de condições complexas de saúde e para a maximização da saúde geral.^(18)^ Tem se mostrado eficaz para várias condições, como cirurgia de emergência e ortopédica; internações; e desfechos de saúde, como quedas, residência em casas de repouso, lesões de pressão, *delirium* e fragilidade física.^(18)^ Por utilizar ferramentas estruturadas,^(13,15)^ a AGA deve ser realizada por um médico experiente e uma equipe multidisciplinar, espelhando o atual modelo assistencial pós-UTI.

Portanto, o processo de AGA pode ser adaptado ao contexto pós-UTI (Figura 1). O uso sistemático da AGA na assistência pós-UTI poderia beneficiar os sobreviventes por meio de vários mecanismos.^(13,15)^ Primeiro, ao empregar ferramentas estruturadas semelhantes às recomendadas para a assistência pós-UTI, a AGA permite uma avaliação abrangente e multidisciplinar de vários domínios. Essa abordagem estruturada facilita a identificação de pacientes frágeis e que possam necessitar de modelos assistenciais alternativos, como transferência para instalações de cuidados pós-agudos ou atenção domiciliar, reabilitação mais intensiva ou aumento da frequência de acompanhamento. Essa abordagem direcionada, também chamada de manejo guiado por avaliação geriátrica, visa minimizar o risco de implementação de tratamentos inadequados e mitigar o potencial de doenças iatrogênicas. Além disso, a integração padronizada e estruturada de informações de múltiplos domínios no processo de AGA contribui para o desenvolvimento de um plano de manejo individualizado.

**Figura 1:** Evolução clínica da fragilidade em pacientes críticos frágeis e não frágeis, com proposta de atuação da avaliação geriátrica ampla no modelo assistencial pós- unidade de terapia intensiva. UTI - unidade de terapia intensiva; AGA - avaliação geriátrica ampla.

Essa estratégia personalizada melhora a qualidade geral da assistência ao abordar as necessidades únicas e complexidades de cada paciente. Além disso, aplicar o processo de AGA para estratificar a assistência pós-UTI com base no estado de fragilidade permite a criação de modelos assistenciais personalizados. Essa abordagem reconhece e inclui diversas condições e necessidades de saúde, melhorando a eficácia e a resposta de intervenções pós-UTI. Ao adaptar o processo de AGA ao contexto pós-UTI, os profissionais de saúde podem melhorar a qualidade da assistência a sobreviventes, minimizando os potenciais resultados negativos associados à PICS.

Em resumo, a implementação sistemática da AGA na assistência pós-UTI tem o potencial de otimizar os desfechos do paciente, ao promover intervenções direcionadas, planos de manejo personalizados e modelos assistenciais adaptados, contribuindo para a melhoria geral da prestação de cuidados após a alta da UTI.
